# Supplementary figures and images for: Detection of vasculogenic mimicry in equine ocular, oronasal, and genital squamous cell carcinoma
Source: PLoS One. 2026 Jan 5;21(1):e0328584. doi: 10.1371/journal.pone.0328584 (PMC12768272; doi:10.1371/journal.pone.0328584)

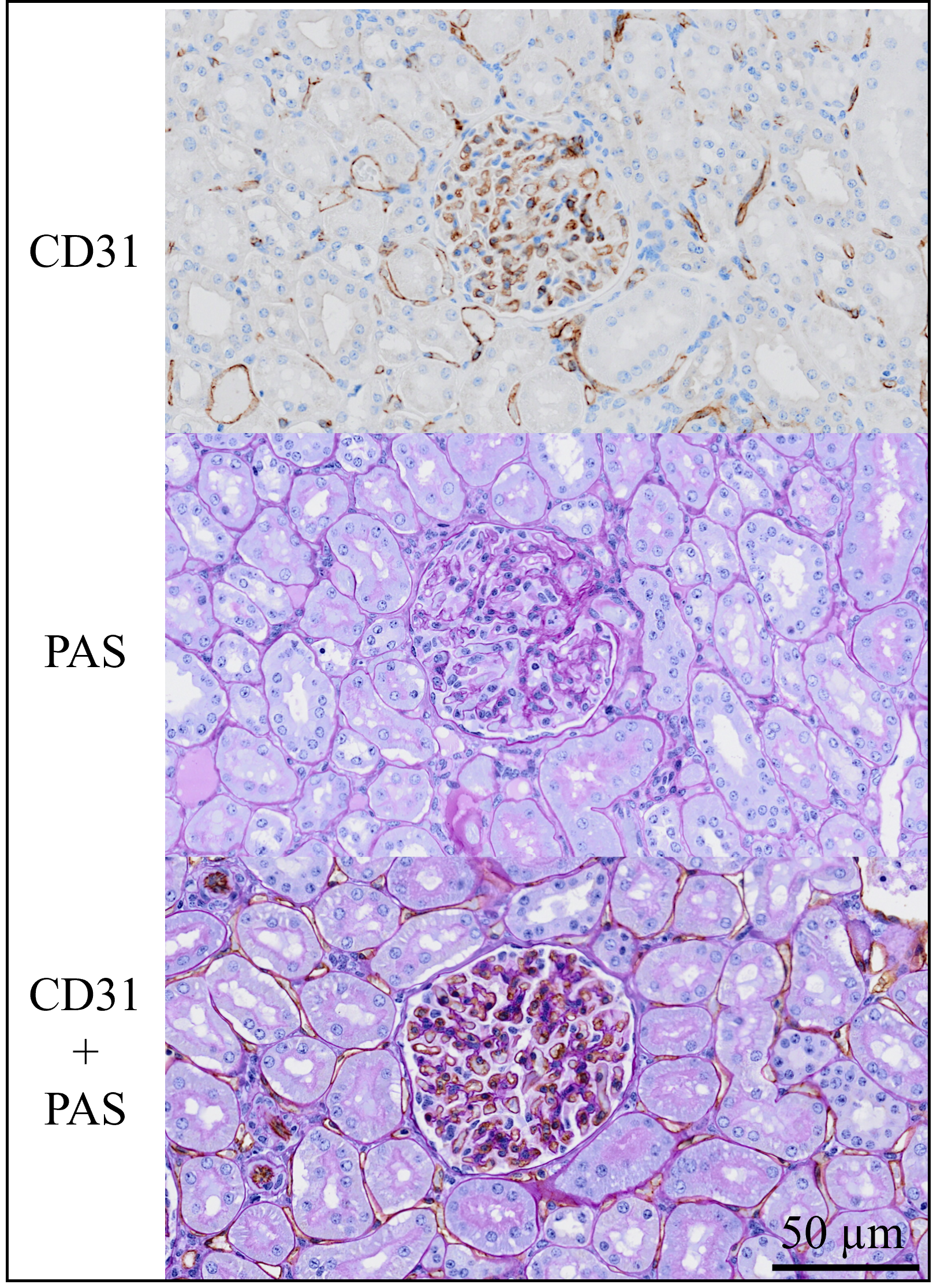

Supplement: S1 Fig — The figure depicts CD31-positive capillaries (brown signal) in equine kidney tissue including a glomerulus. Pas-staining (magenta) highlights the basement membranes of the glomerular capillary loops and the tubular epithelium. Scale bar = 50 µm. (TIF) [file pone.0328584.s001.tif]

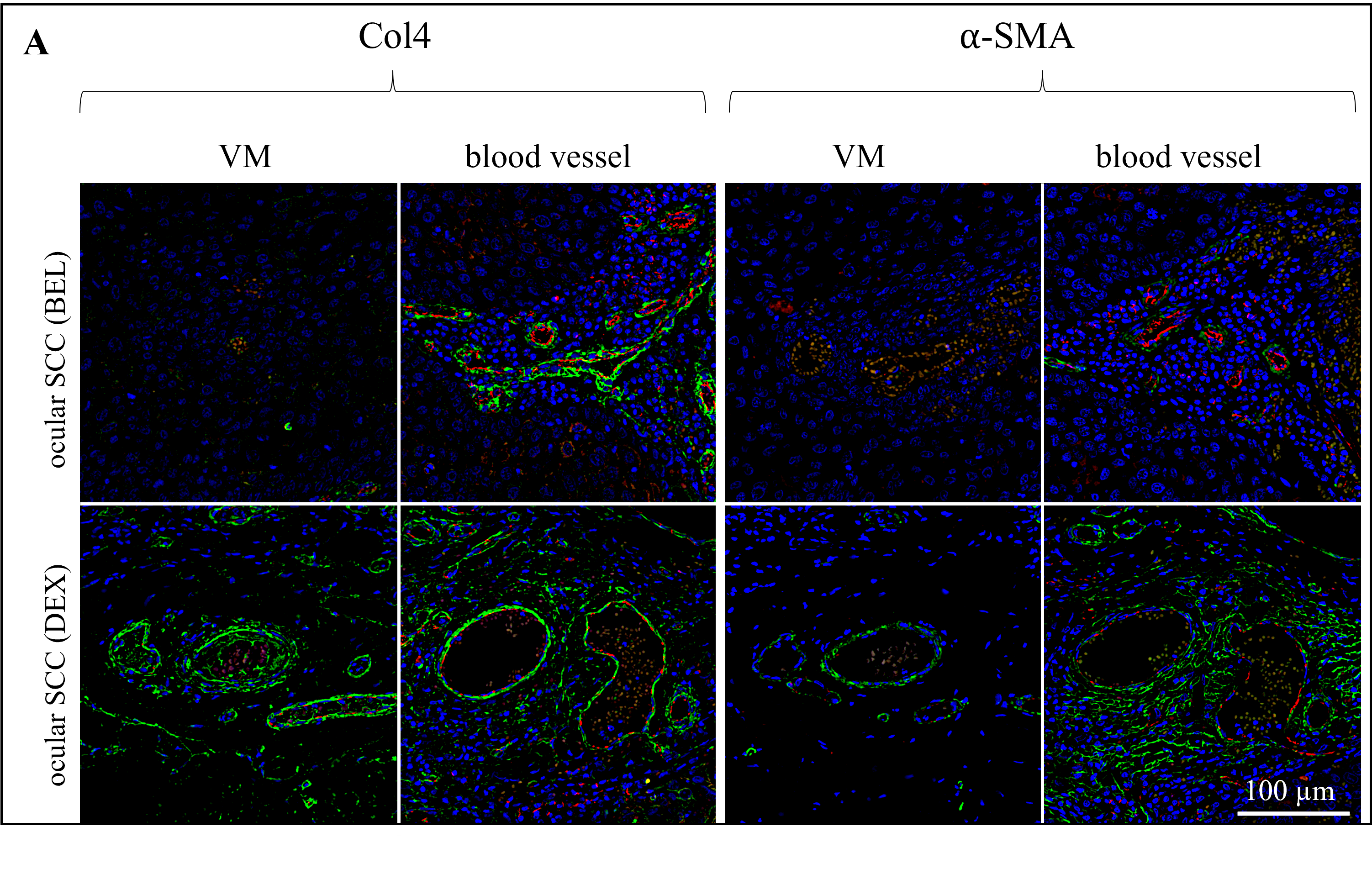

Supplement: S2 Fig — The figure depicts sections of two ocular SCCs (BEL, DEX) that were IF-stained for CD31 (red signal), KRT (orange signal), and Col4 or α-SMA (green signal). The blue signal reflects DAPI-stained cell nuclei. The figures show merged images from normal blood vessels and VM structures for direct comparison. Scale bars = 100 µm. (TIF) [file pone.0328584.s002.tif]

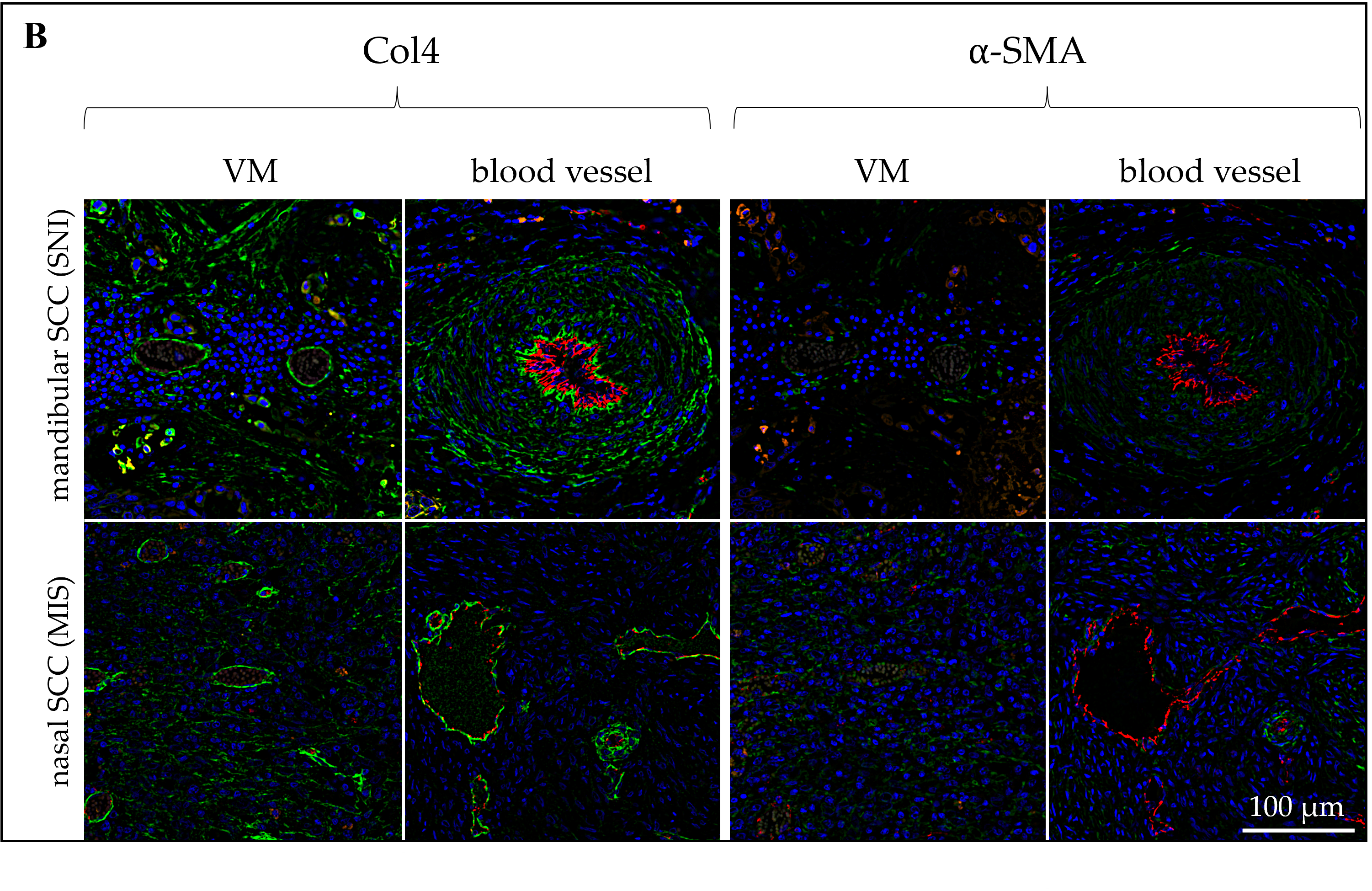

Supplement: S3 Fig — The figure depicts sections of a mandibular (SNI) and a nasal SCC (MIS) that were IF-stained for CD31 (red signal), KRT (orange signal), and Col4 or α-SMA (green signal). The blue signal reflects DAPI-stained cell nuclei. The figures show merged images from normal blood vessels and VM structures for direct comparison. Scale bars = 100 µm. (TIF) [file pone.0328584.s003.tif]

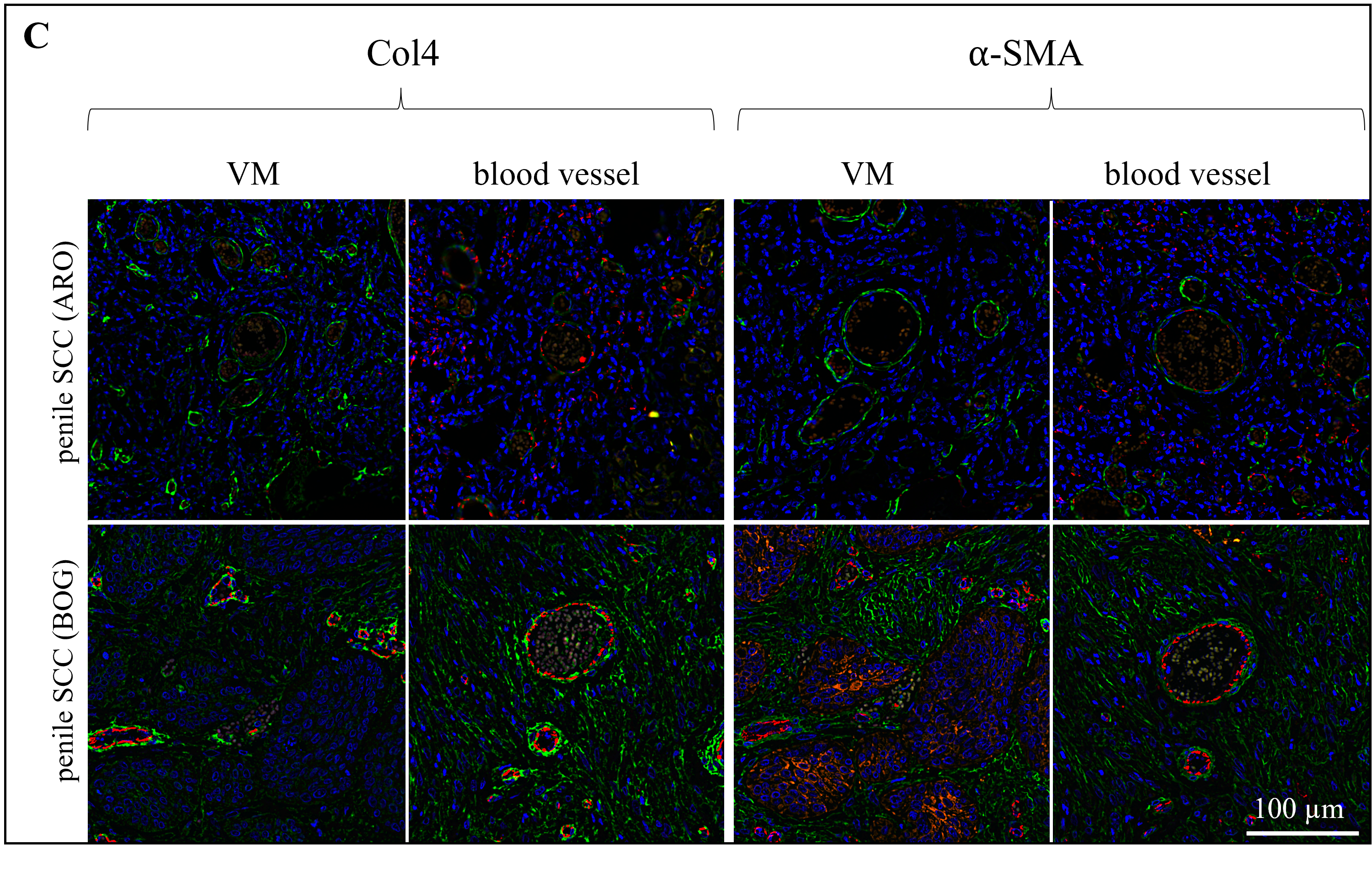

Supplement: S4 Fig — The figure depicts sections of two penile SCCs (ARO, BOG) that were IF-stained for CD31 (red signal), KRT (orange signal), and Col4 or α-SMA (green signal). The blue signal reflects DAPI-stained cell nuclei. The figures show merged images from normal blood vessels and VM structures for direct comparison. Scale bars = 100 µm. (TIF) [file pone.0328584.s004.tif]

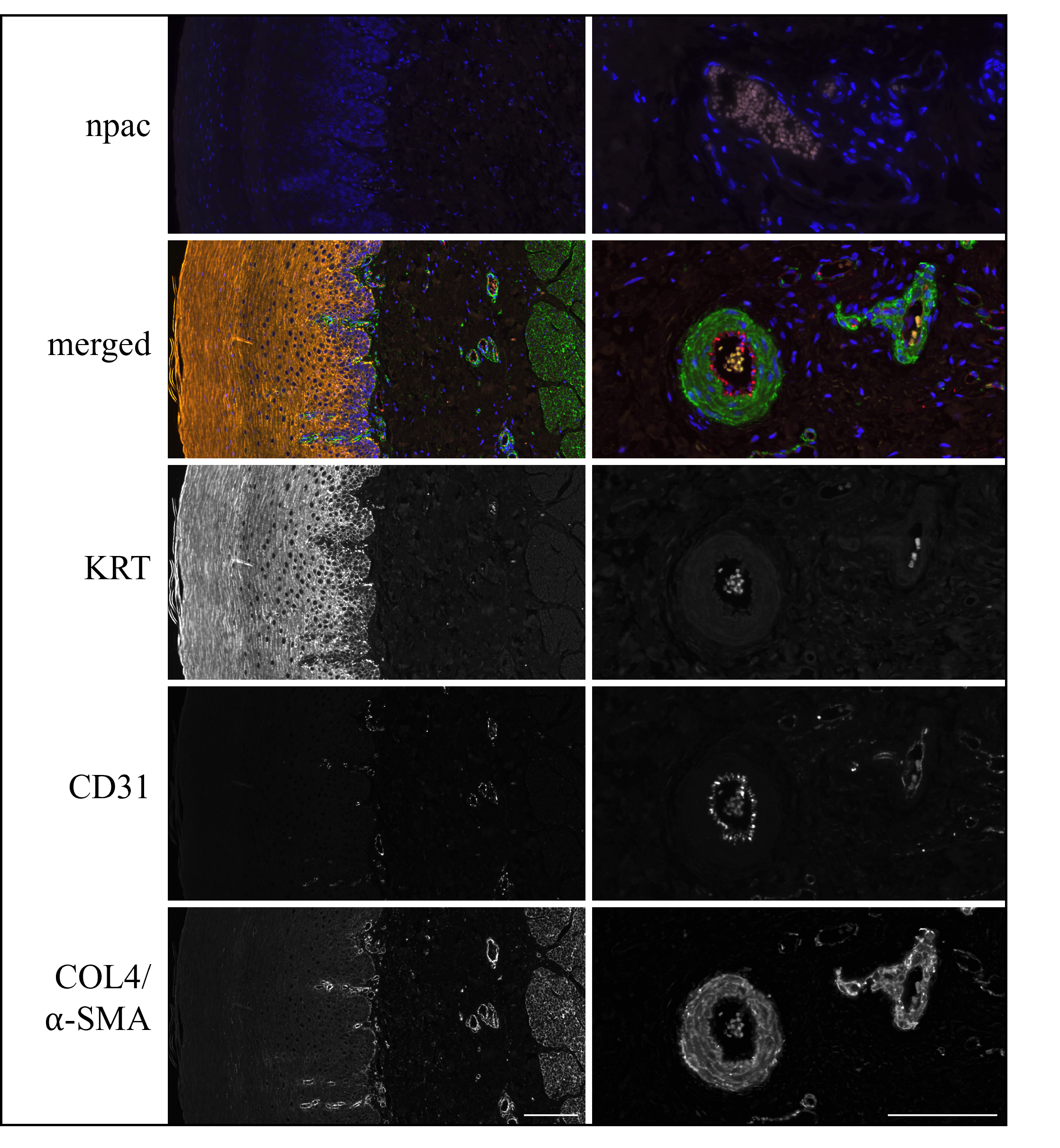

Supplement: S5 Fig — Equine esophagus sections were IF-stained for CD31 (red signal), KRT (orange signal), and Col4 (left side; green signal), or α-SMA (right side; green signal). The figures show merged images and the no-primary-antibody controls in color, and individual staining results in black-and-white. The blue signal reflects DAPI-stained cell nuclei. Left-side-images display the esophageal mucosa lined by squamous epithelium (KRT+; orange signal), the submucosa, and a part of the muscular layer. Ride-side-images show blood vessels in the submucosa at higher magnification. They are lined by CD31+ endothelial cells (red signal) surrounded by the α-SMA+ and Col4+ tunicae (green signals). Scale bars = 100 µm. (TIF) [file pone.0328584.s005.tif]
