# Supplementary material for: Detection of vasculogenic mimicry in equine ocular, oronasal, and genital squamous cell carcinoma
Source: PLoS One. 2026 Jan 5;21(1):e0328584. doi: 10.1371/journal.pone.0328584 (PMC12768272; doi:10.1371/journal.pone.0328584)
Supplement: S1 Table — (DOCX) [file pone.0328584.s006.docx]

| **Supporting information**  **S1 Table:** Horse and disease specifications including information on EcPV and Herpesvirus infection and the VM status | | | | | | |  |  |  |  |
| --- | --- | --- | --- | --- | --- | --- | --- | --- | --- | --- |
| **ID** | **Breed** | **Age** | **Color** | **Sex** | **Diagnosis** | **Remarks** | | **EcPV** | **HV*** | **VM** |
| **Horses with oronasal SCC (n = 14)** | | | | | | | | | | |
| BOM | Warmblood | 24 | bay | M | Lingual SCC (†) | No cornification, poor differentiation of tumor cells, retropharyngeal LN metastases | | N | na | N |
| DAN | WB | 22 | chestnut | G | Nasal SCC (†) | Partial cornification of tumor cells, one retropharyngeal LN metastasis | | **2** | AHV5 | N |
| FLX | Pony | 27 | sorrel | G | SCC of the pharynx, larynx, and trachea (†) | Poorly differentiated, non-cornified tumor cells, LN metastases | | N | na | N |
| JON | Pony | 25 | chestnut | G | SCC of the gingiva, palate, and pharynx (†) | Tumor fills the entire retropharyngeal space, no differentiated, no cornification | | **2** | EHV2 | YES |
| KLU | Noriker | 17 | black hat | G | Maxillary sinus SCC (†) | Moderate cornification of tumor cells, infiltration of the vasculature | | **2** | na | N |
| KRA | Icelandic horse | 21 | chestnut | G | Oropharyngeal SCC (†) | No cornification of tumor cells, retropharyngeal LN metastases | | N | na | N |
| MEC | Shetland | 25 | black | M | SCC of the mandible and maxilla (†) | Tumor causes osteolysis, tumor cells are cornified | | N | na | N |
| MMO | Friesian-Haflinger | 13 | bay | G | SCC nasal cavity, concho-frontal sinus (excised) | Enlarged pleomorphic non-cornified tumor cells, osteolysis | | N | na | YES |
| NEP | Lusitano | 21 | grey | G | SCC of the base of the tongue (†) | Poorly differentiating tumor cells showing hyperchromatic, prominent nuclei | | **2** | EHV+ | N |
| PER | Trotter | 20 | bay | G | Maxillary sinus SCC (†) | Tumor cells partly cornified, osteolysis, retropharyngeal LN metastases | | N | na | N |
| PRI | Shetland | 26 | black | G | SCC sinus, nasal septum (†) | Cornifying tumor | | **2** | N | N |
| SAM | Haflinger-WB | 13 | sorrel | G | SCC of the larynx and pharynx (†) | Partly cornifying SCC, retropharyngeal LN metastases | | **2** | EHV5 | N |
| SNI | Connemara | 16 | grey | M | Mandibular SCC (†) | Metastasizing tumor of non-cornified cells causing marked osteolysis | | **5** | N | YES |
| SIR | WB | 11 | chestnut | M | SCC alveolar process of the incisive bone | Differentiating tumor cells showing dyskeratosis and inverted cornification | | **2** | na | N |
| **Horses with ocular SCC or CIS; (n = 15)** | | | | | | | | | | |
| AMI | Criollo | 12 | paint | G | SCC OS, OD (resected) | Cornifying tumors, horny protrusions | | N | EHV2 | N |
| BEL | Haflinger | 14 | sorrel | M | SCC of the cornea OD (resected) | Partly cornified tumor | | N | N | YES |
| BOD | WB | 19 | chestnut | G | SCC of the nictitating membrane OD (resected) | Poorly differentiated tumor cells with hyperchromatic nuclei | | N | AHV5 | YES |
| JHC | Haflinger | 11 | sorrel | G | Conjunctival CIS OS (resected) | Hyperplastic spinal layer, irregular differentiation at tumor margins, horn pearls | | N | EHV+ | N |
| KIR | Haflinger | 19 | sorrel | M | Limbal SCC OD (resected) | SCC with locally cornifying tumor cell nests | | N | EHV5 | N |
| LAD | Haflinger | 14 | sorrel | M | SCC OD nictitating membrane (resected) | Poorly differentiated, almost not cornifying SCC | | N | N | N |
| MCH | Connemara | 15 | grey | M | Recurrent SCC (†) | Tumor with only locally cornified cells, head and neck metastases | | **2** | EHV2 | YES |
| NAP | Hungarian WB | 24 | chestnut | G | SCC of the nictitating membrane OD (resected) | Poorly differentiated SCC infiltrating the Lamina propria | | N | EHV+ | N |
| NIN | Haflinger | 4 | sorrel | G | Conjunctival SCC OD (resected) | Invasively growing tumor | | N | N | N |
| TKB | Irish Cob | 8 | black | M | SCC OS nictitating membrane (resected) | Poorly differentiated non-cornifying tumor cells also clustering in small vessels | | N | na | N |
| TSU | Haflinger | 6 | sorrel | M | SCC OD nictitating membrane (resected) | Non-cornified, extensive lesion invading and destroying the Cornea | | N | na | N |
| MXA | Haflinger | 10 | sorrel | M | SCC cornea, limbus, conjunctiva OD (resected) | Poorly differentiating/cornifying tumor cells, proliferates through basal membrane | | N | na | N |
| DEX | Trakehner | 16 | piebald | G | SCC OD nictitating membrane (resected) | Exophytic, non-cornified SCC in situ 3^rd^ eyelid | | N | EHV2 | YES |
| SUS | Friesian horse | 15 | black | M | SCC OD conjunctiva (resected) | Strongly proliferating tumor penetrating the basal membrane | | N | na | N |
| SIS | Noriker | 10 | spotted | M | SCCs nictitating membrane, cornea (resected) | Strongly proliferating tumor penetrating the basal membrane, medium cornification | | N | na | N |
| **Horses with genital SCC or CIS; (n = 14)** | | | | | | | | | | |
| ARO | Hanoverian | 18 | bay | G | Penile SCC (†) | Metastatic tumor, non-cornified, poorly differentiated cells inguinal LN metastases | | **2** | EHV2 | YES |
| BAK | Icelandic horse | 27 | chestnut | G | Recurrent SCC (†) | Cornifying lesion displaying horn pearls, inguinal LN metastases | | **2** | N | N |
| BIK | Icelandic horse | 30 | chestnut | G | Penile SCC (†) | Late stage SCC with LN and lung metastases | | **2** | EHV2 | N |
| BLÁ | Icelandic horse | 23 | chestnut | G | Penile SCC (penile en-bloc resection) | Proliferative, well differentiated cells, some protrusions into subepithelial tissue | | **2** | N | YES |
| BOG | Hungarian WB | 28 | black | G | Penile SCC (†) | Complete organ destruction, horn pearls, inguinal LN metastases | | **2** | N | YES |
| BRY | Icelandic horse | 24 | black | G | Penile SCC (penile en-bloc and LN resection) | Cornifying tumor of the Glans penis | | **2** | EHV2 | N |
| ERC | WB | 15 | bay | G | SCC glans penis (partial penile resection) | Tumor of non-cornified tumor cells, partially protruding through basal membrane | | **2** | N | YES |
| GJE | WB | 20 | skewbald | M | Vulval SCC (resection) | Moderately differentiated cells with hyperchromatic nuclei | | **2** | N | YES |
| JAC | Haflinger | 17 | sorrel | G | Penile SCC (†) | Cornified tumor of the Glans penis with no evidence of metastases | | **2** | N | N |
| MEL | Cob | 15 | piebald | G | Penile SCC (penile en-bloc resection) | Non-cornified tumor protruding through basal membrane | | **2** | EHV2 | YES |
| SHN | Criollo | 18 | skewbald | G | Penile CIS (resected) | Precancerous neoplasia with an intact basal membrane | | **2** | AHV5 | N |
| STA | Haflinger | 15 | sorrel | G | Penile SCC (penile en-bloc resection) | Tumor of the Glans penis displaying horn pearls | | **2** | EHV2 | N |
| TAL | WB | 23 | bay | G | Penile SCC | Cornifying SCC invading regional LNs | | **2** | AHV5 | N |
| TVI | Icelandic horse | 27 | bay | G | Penile SCC (penile en-bloc resection) | Superficial SCC of the Fossa glandis, LNs are tumor-free | | **2** | EHV5 | N |

*HV: Herpesvirus infection status according to Miglinci, L., Reicher, P., Nell, B., Koch, M., Jindra, C., and Brandt, S. (2023). Detection of equine papillomaviruses and gamma-herpesviruses in equine squamous cell carcinoma. Pathogens 12, 179. https://doi.org/10.3390/pathogens12020179

ID: horse identification code; Age: in years; G: gelding (castrated male); M: mare (female); SCC: squamous cell carcinoma; CIS: carcinoma in situ; LN: lymph node; OD: oculus dexter, right eye; OS: oculus sinister, left eye; in parentheses: outcome, with † designating euthanization; N: Not detected; na: not assessed.
